# Copper restriction unmasks axonal degeneration in a mouse model of X-linked hereditary motor neuropathy

**DOI:** 10.1093/mtomcs/mfag020

**Published:** 2026-06-05

**Authors:** G Perez-Siles, M Ellis, G J Song, D P Gupta, M Khalil, M Damjancuk, S La Fontaine, M L Kennerson

**Affiliations:** School of Medical Sciences, Faculty of Medicine and Health, University of Sydney, Sydney, NSW 2050, Australia; Northcott Neuroscience Laboratory, ANZAC Research Institute, Sydney, NSW 2139, Australia; Northcott Neuroscience Laboratory, ANZAC Research Institute, Sydney, NSW 2139, Australia; Translational Brain Research Center, International St. Mary’s Hospital, Catholic Kwandong University, Incheon 22711, Republic of Korea; Department of Medicine, College of Medicine, Catholic Kwandong University, Gangneung, Gangwon-do 26501, Republic of Korea; Translational Brain Research Center, International St. Mary’s Hospital, Catholic Kwandong University, Incheon 22711, Republic of Korea; Molecular Physiology Unit, ANZAC Research Institute, Sydney, NSW 2139, Australia; Molecular Physiology Unit, ANZAC Research Institute, Sydney, NSW 2139, Australia; School of Life and Environmental Sciences, Deakin University, Burwood, VIC 3125, Australia; The Florey Neuroscience Institute, University of Melbourne, Parkville, VIC 3052, Australia; School of Medical Sciences, Faculty of Medicine and Health, University of Sydney, Sydney, NSW 2050, Australia; Northcott Neuroscience Laboratory, ANZAC Research Institute, Sydney, NSW 2139, Australia; Molecular Medicine Laboratory, Concord Repatriation General Hospital, Sydney, NSW 2139, Australia

## Abstract

Mutations in the copper (Cu) transporter *ATP7A* cause a spectrum of X-linked diseases, including Menkes Disease, Occipital horn syndrome, and distal hereditary motor neuropathy (dHMNX). We previously generated a conditional knock-in mouse model of dHMNX expressing Atp7a^T985I^, the murine orthologue of the human T994I variant identified in dHMNX patients. Although A*tp7a^T985I^* mice did not develop overt motor degeneration, affected males showed a trend toward reduced Cu levels in the peripheral nervous system (PNS). The high-affinity copper transporter Ctr1, encoded by *Slc31a1*, regulates Cu uptake, and ubiquitous heterozygosity for *Slc31a1* (*Ctr1*^+/−^) has been reported to limit Cu availability in the nervous system without impairing motor performance. In this study, we genetically restricted Cu availability in *Atp7a^T985I^* mice by crossing them with *Ctr1^+/−^* animals. *Atp7a^T985I^/Ctr1^+/−^* males exhibited significantly reduced Cu levels in both the central nervous system and PNS compared to wild-type littermates. At 6 months of age, behavioural testing and histopathological assessment revealed mild motor deficits and axonal loss, preferentially affecting small-caliber fibres exclusively in the Cu-restricted *Atp7a^T985I^*males. Tandem mass tag-based proteomics of sciatic nerve identified significant changes linked to energy metabolism, cytoskeletal integrity, and cellular stress responses. Together, these data show that limiting Cu availability unmasks a dHMNX-like phenotype in *Atp7a^T985I^/Ctr1^+/−^* mice, demonstrate the critical role of Cu availability in maintaining peripheral axons and suggest that reduced Cu in motor neurons contributes to axonal degeneration in dHMNX.

## Graphical Abstract

**Graphical Abstract:**
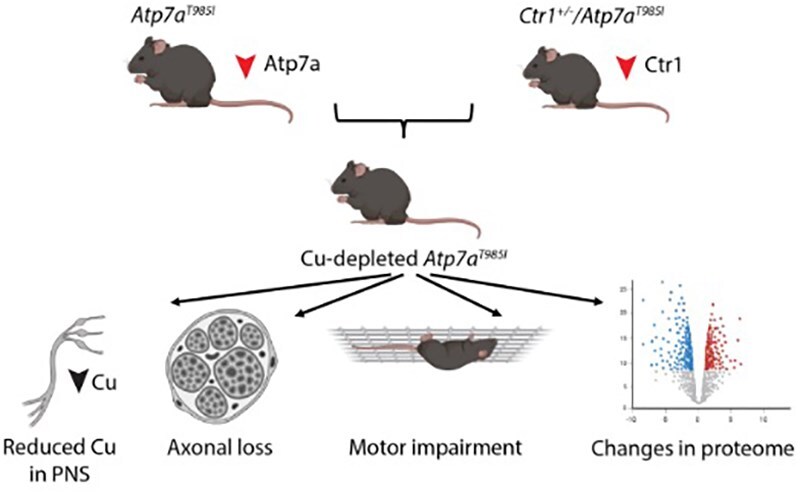

## Introduction

Copper (Cu) is an essential trace element that plays a pivotal role in numerous biological processes. Due to its key role in oxidative metabolism, antioxidant defence, and neurotransmitter synthesis, Cu is particularly important for neuronal development and sustaining neuronal function (reviewed in [1]). The importance of Cu in the nervous system is underscored by the severe clinical phenotypes observed in disorders in which Cu dysregulation constitutes a key pathological process, such as Menkes disease (MD), and by accumulating evidence suggesting a role for altered Cu homeostasis in neurodegenerative conditions including motor neuron disease [2] and Parkinson’s disease [3].

ATP7A is a P_1B_-type copper ATPase that maintains Cu homeostasis by exporting Cu from cells and delivering Cu to cuproenzymes within the secretory pathway [4]. Loss-of-function mutations in *ATP7A* cause systemic Cu deficiency disorders, including MD [5–7] and occipital horn syndrome (OHS) [8]. In contrast, specific missense variants (A991D, T994I, and P1386S) cause X-linked distal hereditary motor neuropathy (dHMNX), a nonfatal neurodegenerative condition marked by length-dependent axonal degeneration in the absence of overt systemic Cu deficiency [9, 10].

Understanding the mechanistic and pathophysiological basis of dHMNX remains challenging in part because no animal model yet fully recapitulates the key disease processes occurring in affected patients. A mouse model in which the *Atp7a* gene was selectively knocked out in motor neurons led to a degenerative phenotype and provided further evidence for the importance of Cu in the maintenance and function of motor neurons [11]. However, the complete ablation of Atp7a observed in this model is unlikely to reflect the biology of dHMNX, where disease-causing missense variants are thought to retain partial ATP7A activity [9, 12]. As a result, knock-out models may miss the subtle cellular dysfunction that precedes and drives the underlying axonal degeneration in dHMNX. We previously reported a conditional knock-in mouse model expressing the murine orthologue (*Atp7a^T985I^*) of the dHMNX-associated ATP7A T994I mutation [13]. This model recapitulates key molecular features observed in patient fibroblasts and patient iPSC-derived motor neurons, including reduced ATP7A protein abundance and disrupted Cu homeostasis [12]. While *Atp7a^T985I^*mice did not develop an overt degenerative motor phenotype, they displayed altered Cu levels within the nervous system (including a partial reduction in sciatic nerve Cu) and evidence of abnormal muscle physiology, consistent with a presymptomatic dHMNX state. We therefore hypothesized that further limiting Cu availability within the peripheral nervous system (PNS) of *Atp7a^T985I^*mice may amplify the cellular and molecular consequences associated with the *ATP7A* missense variant and enable development of a symptomatic dHMNX mouse model.

Cellular uptake of Cu in mammals is mediated primarily by the high-affinity Cu transporter CTR1, encoded by *SLC31A1*. Mice heterozygous for *Slc31a1* (*Ctr1^+/−^*) show tissue-selective Cu depletion confined to the central nervous system (CNS) without overt behavioural impairment [14]. In this study, we have genetically restricted Cu availability to the nervous system in *Atp7a^T985I^* mice by crossing *Ctr1*^+/−^ males with females heterozygous for the Atp7a dHMNX allele to generate *Atp7a^T985I^/Ctr1^+/−^* males. We then performed comprehensive behavioural, histopathological, and molecular phenotyping of this model. Genetic restriction of Cu access unmasked a degenerative phenotype, characterized by declining motor performance and axonal pathology in the PNS, supporting a critical requirement for Cu availability in maintaining axonal integrity in dHMNX.

## Results

**Figure 1:**
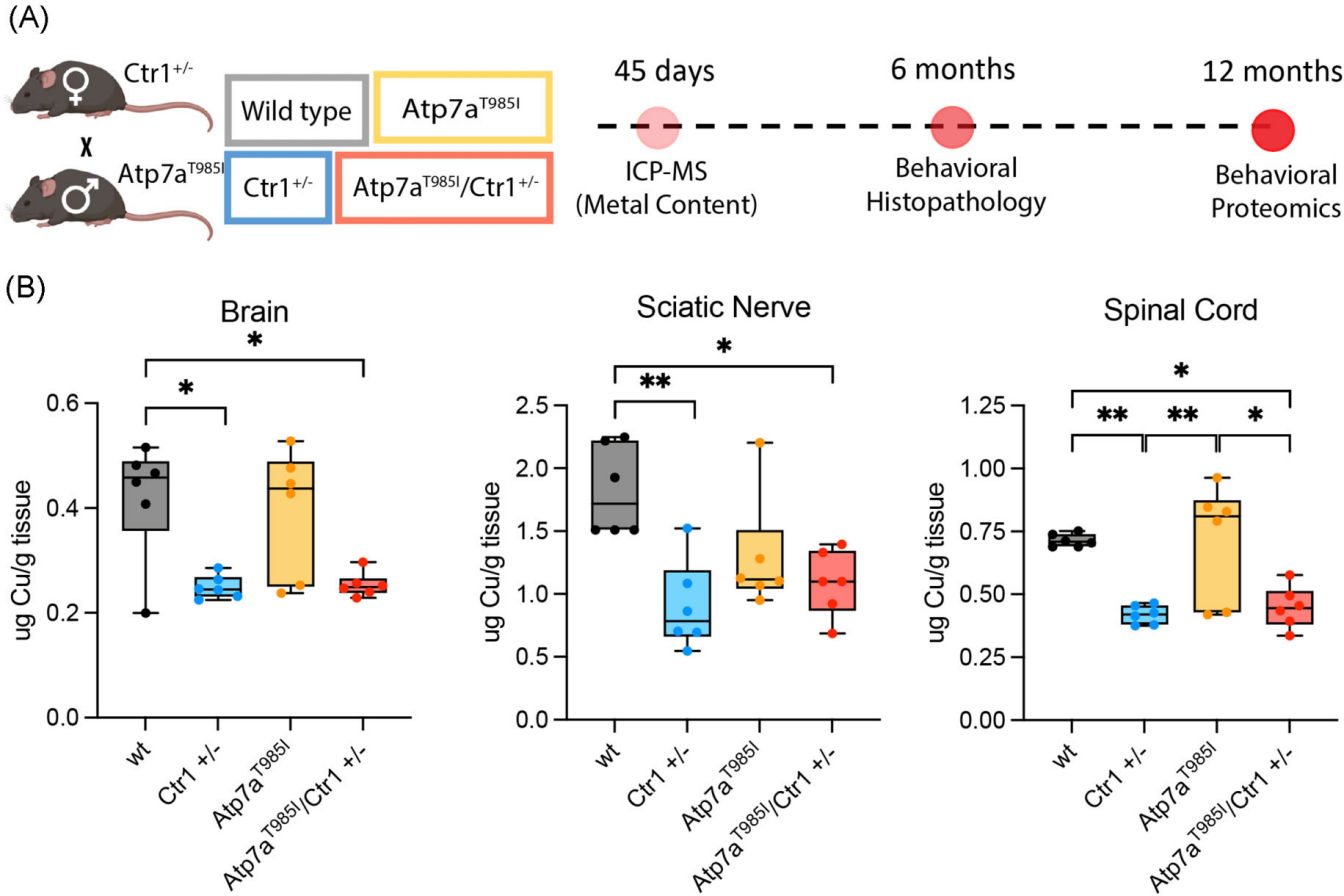
Generation of Cu-restricted *Atp7a^T985I^* males and experimental design. (A) Breeding scheme used to generate compound mutant males. *Ctr1*^+/−^ males were crossed with *Atp7a^T985/+^* females to produce four male genotypes: wild type (wt), Ctr1^+/−^, *Atp7a^T985I^* (hemizygous), and *Atp7a^T985I^/Ctr1^+/−^*. Study timeline: Cu quantification by ICP–MS at 45 days, motor behaviour and sciatic nerve histopathology at 6 months, and motor behaviour and sciatic nerve proteomics at 12 months. (B) Copper concentration in brain, sciatic nerve, and spinal cord from the four genotypes by ICP–MS and expressed as μg of Cu/g tissue. Points represent individual mice (*n* = 6 per genotype). Box plots show the median and interquartile range. Brackets denote significant pairwise comparisons. *P*-values illustrate significant differences between the groups indicated and were obtained from a 2-way ANOVA test (Tukey’s multiple comparisons; *P < .05; **P < .005).

The T994I missense variant in the copper transporter ATP7A is one of the three confirmed pathogenic mutations associated with dHMNX [9]. A conditional knock-in mouse model expressing the murine orthologue (*Atp7a^T985I^)* is currently the only available *in vivo* model for this disease [13], however, *Atp7a^T985I^* mice do not develop overt axonal degeneration or a clear motor phenotype. To test whether limiting Cu availability to the PNS could unmask a symptomatic dHMNX phenotype, we generated compound mutant *Atp7a^T985I^/Ctr1^+/−^* males by crossing *Atp7a^T985I^* heterozygous females with *Ctr1^+/−^* males (Fig. 1A). This breeding strategy produced four experimental genotypes: wild type (wt), *Ctr1^+/−^, Atp7a^T985I^*, and Atp7a^T985I^/Ctr1^+/−^. Since dHMNX is X-linked and predominantly affects males, only male offspring were analysed. The study design (Fig. 1A) comprised metal content quantification (45 days), peripheral nerve histopathology at 6 months, proteomics at 12 months, and behavioural assessments across all genotypes at 6 and 12 months of age.

Consistent with prior reports, *Ctr1^+/−^* animals have reduced total Cu content specifically in the brain [14] and spinal cord [15]. Here we show for the first time that Ctr1 also influences Cu availability in the PNS (Supplementary Fig. 1).

Tissue Cu levels in brain, spinal cord, and sciatic nerve were measured by ICP–MS in 45-day-old males. This experiment shows that our strategy successfully achieved genetic reduction of Cu in the CNS (brain and spinal cord) and PNS (sciatic nerve) of the double mutant (Fig. 1B), hence generating a Cu-restricted *Atp7a^T985I^*model (*Atp7a^T985I^/Ctr1^+/−^*).

**Figure 2:**
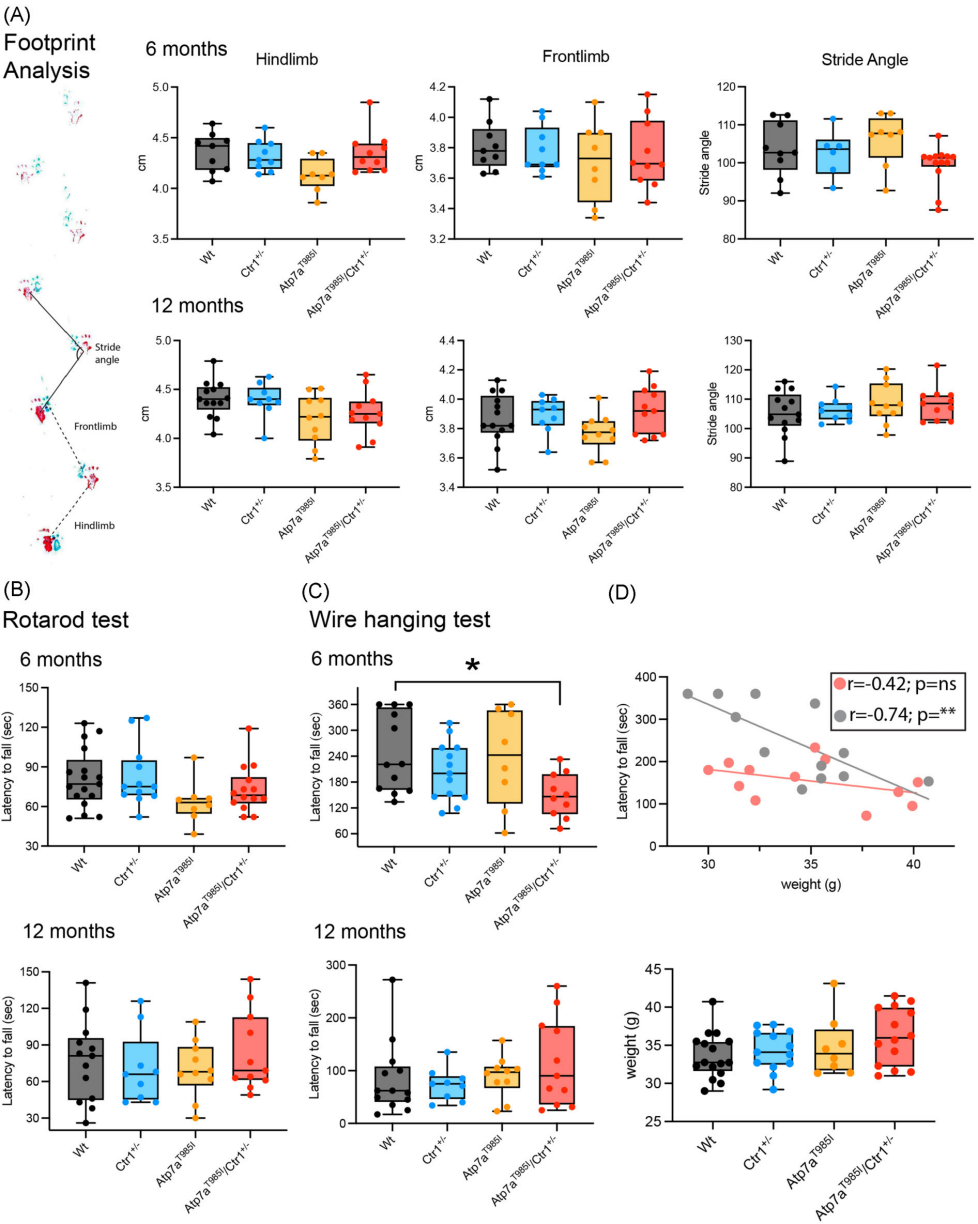
Motor performance in Cu-restricted *Atp7a^T985I^* mice at 6 and 12 months. (A) Footprint gait analysis. Mice traversed an 80 cm runway after fore- and hind-paw inking (schematic left). Front- and hind-limb stride length (cm) and hind paw angle (°) (left schematic) were quantified and recorded for each genotype at 6 months (*n* = 8–10) and 12 months (*n* = 9v13). Individual values are shown overlaid on box and whisker plots. (B) Rotarod performance. Motor co-ordination/endurance was assessed on an accelerating rotarod (4–40 rpm over 300 s). The longest latency to fall (s) from three trials per mouse was analysed for each genotype at 6 months (*n* = 8–17) and 12 months (*n* = 9–13). (C) Inverted wire hanging test. Forelimb/hindlimb strength and endurance were measured as the latency to fall (s) from an inverted metal grid, with a maximum cut-off of 360 s. Each mouse completed three trials separated by ≥5 min rest for 6-month (*n* = 8–13) and 12-month (*n* = 9–13) animals. Statistical significance was assessed by 1-way ANOVA within each age followed by Tukey’s multiple comparisons test (*P < .05). (D) Body weight and wire-hang performance. Scatter plot shows the relationship between wire-hang latency (s) and body weight (g) at 6 months for wt (grey) and Atp7a^T985I^/Ctr1^+/−^ (red) mice. Lines indicate simple linear regression; association was assessed using Pearson’s correlation coefficient (r).c

We then sought to determine whether Cu-restricted *Atp7a^T985I^*mice show motor abnormalities by performing a series of behavioural tests for motor performance (footprint analysis, rotarod test, and wire hanging test) (Fig. 2A-C). Our experiments confirmed data from our previous studies [13] and showed no impaired motor function in the *Atp7a^T985I^*males. These tests also verified that, despite the significantly fewer number of ventral α-motor neurons reported in *Ctr1*^+/−^ animals [15], reduction of Cu in the nervous system is not sufficient to trigger motor dysfunction. Our results demonstrated that *Atp7a^T985I^/Ctr1^+/−^* animals showed a statistically significant reduction in the latency to fall in the wire hanging test at 6 months old (Fig. 2C), indicating the grip strength is affected in the Cu-restricted *Atp7a^T985I^* mice at that age. In wild type mice, body weight was strongly and inversely correlated with inverted wire latency (*r* = −0.74; *P* = .006), indicating heavier animals fell sooner with this behavioural test (Fig. 2D). In *Atp7a^T985I^/Ctr1^+/−^* mice, latency was reduced overall, and was not significantly correlated with weight (*r* = −0.42, *P* = ns), suggesting that the performance deficit cannot be explained by weight alone. Although this phenotype was not observed in 12-month-old males, we attribute this result in older mice to the overall increase in the animals’ weight for all genotypes (mean weight per genotype, wt = 42.5; Ctr1^+/−^=41.9, *Atp7a^T985I^*= 40.8, and Atp7a^T985I^/Ctr1^+/−^=38.7 g) that leads to a profound reduction in the latency to fall for all genotypes.

**Figure 3:**
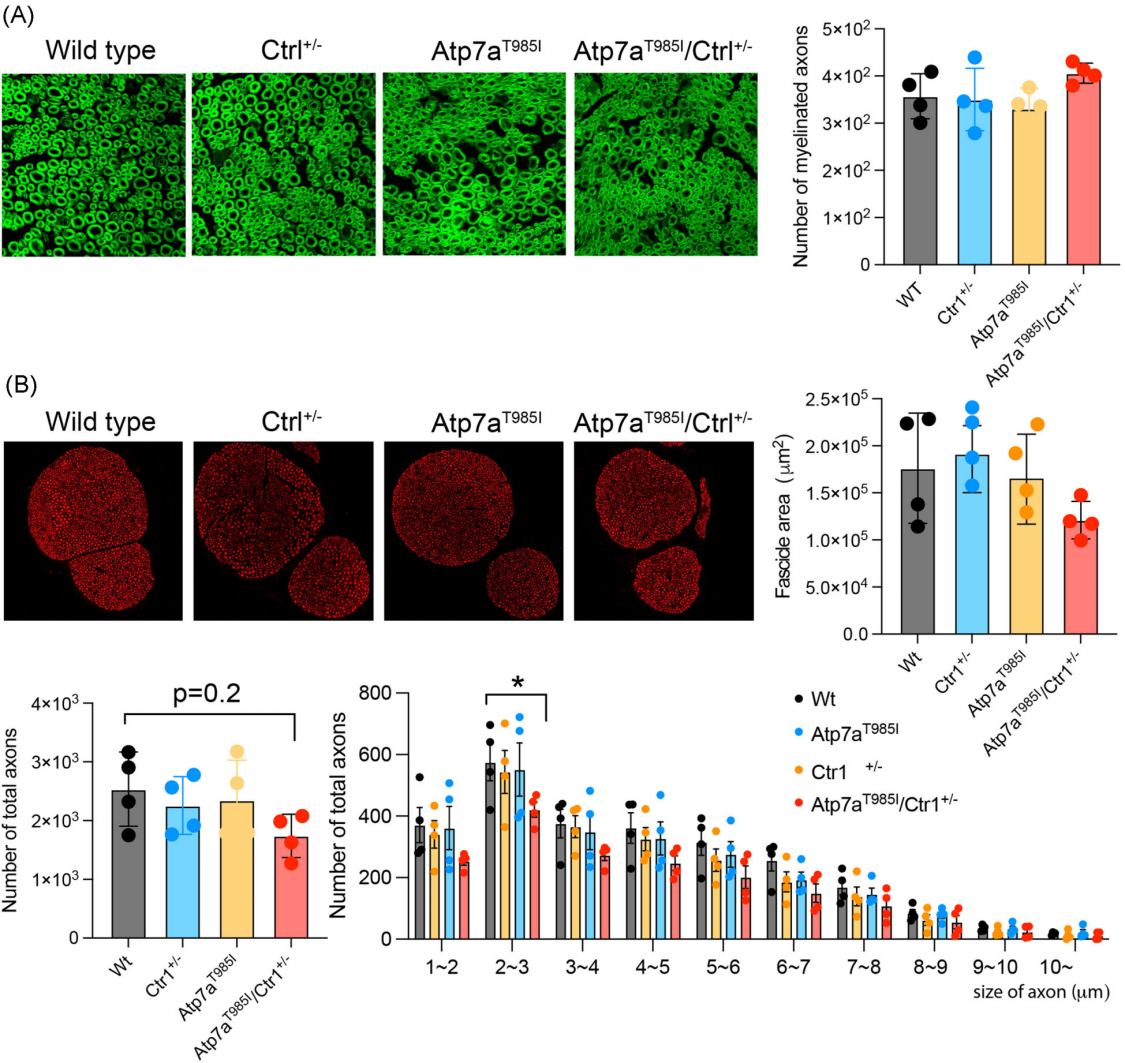
Sciatic nerve histopathology in Cu-restricted *Atp7a^T985I^* model at 12-months of age. (A) Quantitation of myelinated axons sciatic nerve cross-sections stained with fluoromyelin. Each data point represents the mean myelinated axon count per animal calculated from 16 to 26 fields of view (*n* = 4 mice per genotype). (B) Neurofilament immunohistochemistry of sciatic nerve cross sections with quantification of main fascicle area (μm^2^) and total axon number per fascicle (axons >1 μm diameter). Atp7a^T985I^/Ctr1^+/−^ mice show a reduced total axon count relative to the three other genotypes, with a significant deficit in the 2–3 μm axon diameter bin. *P*-values were determined by 2-way ANOVA with Tukey’s multiple comparisons test; *P < .05).

dHMNX is characterized by length-dependent degeneration of distal peripheral axons. To assess whether axonal loss occurs in Cu-restricted *Atp7a^T985I^*mice, semi-thin transverse sections of sciatic nerve from 12-month-old animals were analysed (Fig. 3). Histopathological assessment included quantification of myelinated axon density (Fig. 3A), and calculation of total axon number (Fig. 3B). Consistent with prior observations, fluoromyelin staining revealed no differences in myelinated axon density across genotypes. In contrast, *Atp7a^T985I^/Ctr1^+/−^* males showed a reduction in the fascicle area and total axon number relative to wild-type animals, although these differences did not reach statistical significance (*P* = .23 and *P* = .20, respectively). Notably, stratifying axons by diameter (1–10 μm, binned in 1 μm intervals) revealed a significant loss of small-caliber fibres, with the 2–3 μm population selectively reduced only in the Cu-restricted *Atp7a^T985I^*mice.

**Figure 4:**
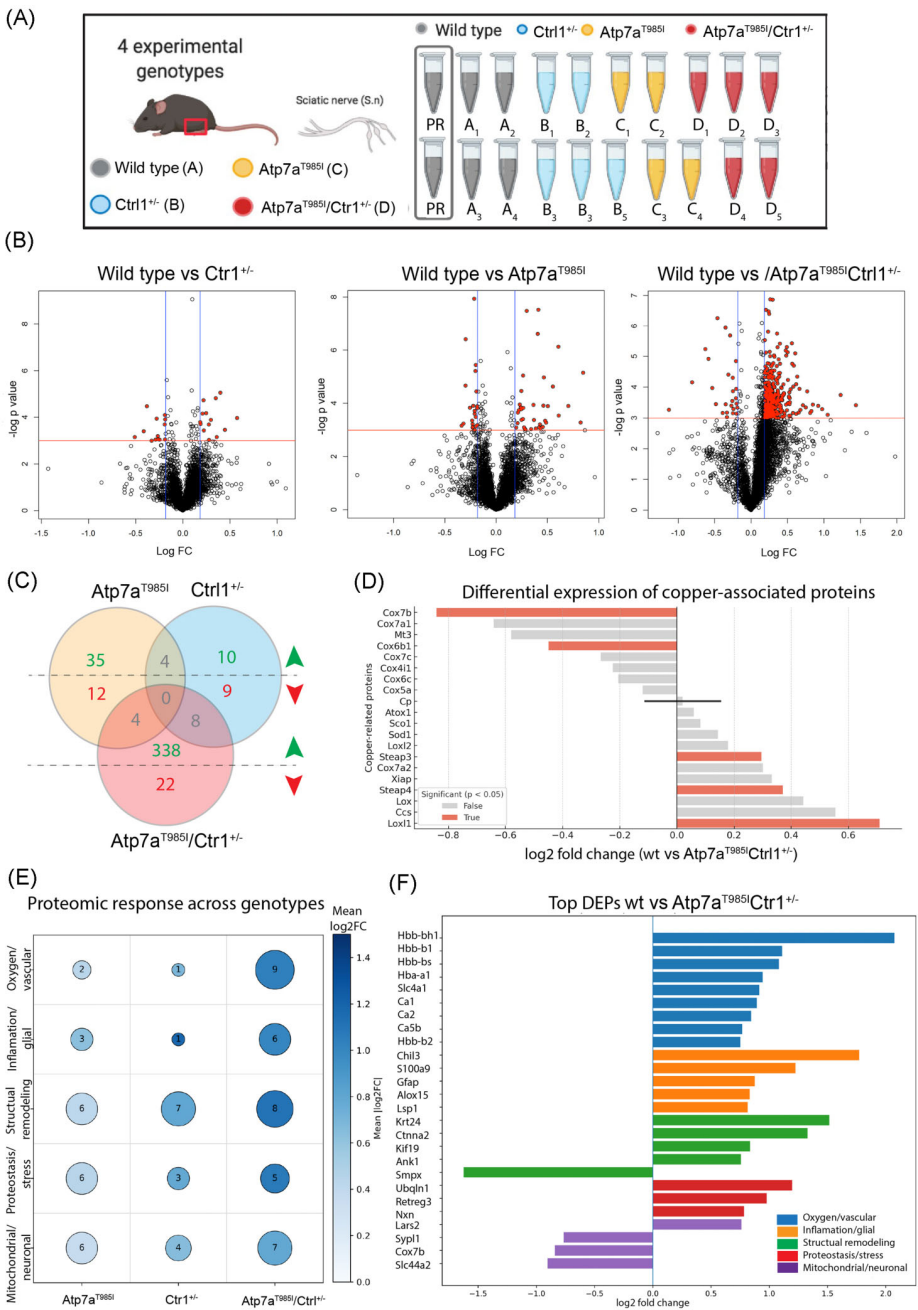
TMT-based quantitative proteomic analysis of sciatic nerves of Cu-restricted Atp7a^T985I^ mouse at 12 months (A) Experimental design using TMT labelling scheme. Sciatic nerves were collected from 12-month-old males from four genotypes with *n* = 4–5 samples per group (A_1-4_: wild type; B_1-5_: Ctr1^+/−^; C_1-4_: Atp7a^T985I^; D_1-5_: Atp7a^T985I^/Ctr1^+/−^). Samples were analysed using two TMT-10-plex batches each including a pooled reference (PR) channel used for between-batch normalization (pooling an equal amount of the 18 samples). (B) Differential protein abundance. Proteomics identified 4808 proteins, of which 3224 proteins were quantified across all samples. Volcano plots show comparisons of *Ctr1^+/−^, Atp7a^T985I^*, and *Atp7a^T985I^/Ctr1^±^* relative to wild type. Each point represents one protein; X-axis represents the log fold change (Log FC); *y*-axis the—log *P*-value. Horizontal red line indicates the *P*-value threshold (*P* < .05) and vertical blue lines indicate the fold-change cut-off (±1.2-fold). Proteins meeting both criteria are highlighted in red. (C) Overlap of DEPs across genotypes. Venn diagram summarizes DEPs unique and shared among *Ctr1^+/−^, Atp7a^T985I^*, and *Atp7a^T985I^/Ctr1^+/−^* relative to wt. Numbers in green indicated upregulated DEPs and numbers in red denote downregulated DEPs. (D) Cu-related proteins. Bar plot shows the log₂ fold change of selected Cu associated proteins in the *Atp7a^T985I^/Ctr1^+/−^* group vs wild-type; bars are coloured by statistical significance (red: significant, *P* < .05; grey: nonsignificant). Negative values indicate reduced abundance in the Cu-restricted genotype. (E) Bubble plot showing DEPs identified from pairwise comparisons of sciatic nerve proteomes (*Atp7a^T985I^, Ctr1*^+/−^, and *Atp7a^T985I^*/*Ctr1*^+/−^ versus wt). Bubble size reflects the number of DEPs assigned to each functional category based on curated annotation (oxygen/vascular processes, inflammation/glial activation, structural remodelling, proteostasis/stress responses, and mitochondrial/neuronal function). Colour intensity represents the mean absolute log2 fold change within each category. (F) Bar graph showing top DEPs (ranked by absolute log2 fold change, |log2FC| > 0.75) identified from the pairwise comparison between *Atp7a^T985I^*/*Ctr1*^+/−^ and wild type (wt) sciatic nerve (*P* < .05, unadjusted Student’s *t*-test), grouped into functional categories. Bar length represents log2 fold change (positive values indicate upregulation; negative values indicate downregulation).

To gain insight into the molecular basis of dHMNX pathogenesis, we performed quantitative proteomic profiling of sciatic nerves. Sciatic nerves were collected from 12-month-old mice (*n* = 4–5 per genotype), proteins were extracted and digested, and peptides were labelled with tandem mass tags (TMTs) for multiplexed quantification (Fig. 4A). In total, 4808 proteins were identified, of which 3224 were quantified across all biological replicates (full dataset in Additional file 1: Table S1). To maximize sensitivity and identify coordinated biological patterns, we employed a discovery-oriented analytical approach using unadjusted *P*-values (Student’s t-test, *P* < .05; fold-change > 1.2). Using this strategy, we detected 19 and 47 proteins that were differentially expressed (hereafter referred to as DEPs) in *Ctr1*^+/−^ and *Atp7a^T985^* mice, respectively, relative to wild-type animals (Fig. 4B). The most pronounced changes were observed in the Cu-restricted *Atp7aT^985I^/Ctr1*^+/−^ model, with 360 DEPs predominantly upregulated (338 up vs. 22 down; Fig. 4C), indicating a marked genotype-dependent shift in the sciatic nerve proteome. To unbiasedly identify biological pathways perturbed in Cu-restricted *Atp7a^T985I^* nerves, we performed Gene Ontology (GO) enrichment analysis (Supplementary Fig. 2). DEPs in *Atp7a^T985I^/Ctr1^±^* versus wild type were significantly enriched for terms related to *protein-containing complex assembly, metabolic/catabolic process*, and *transport*. This was consistent with broad remodelling of protein homeostasis and energy-related pathways under conditions of restricted Cu availability.

Given the central role of copper in neuronal metabolism, we examined proteins directly involved in Cu handling and Cu-dependent processes (Fig. 4D). Proteins involved in Cu homeostasis were largely unchanged in *Ctr1*^+/−^ and *Atp7a^T985I^* sciatic nerves, consistent with previous transcriptomic analyses in this model. In contrast, *Atp7a^T985I^*/*Ctr1*^+/−^ nerves showed selective dysregulation of Cu-related proteins, including downregulation of cytochrome c oxidase subunits (Cox7b, Cox6b1) and upregulation of Cu-linked proteins (Steap3, Steap4, Loxl1). Increased expression of metalloreductases Steap3 and Steap4 may reflect a compensatory response aimed at enhancing cellular metal uptake under conditions of limited Cu availability. Conversely, reduced abundance of Cox subunits, key components of mitochondrial complex IV, suggests impaired oxidative phosphorylation, consistent with the observed enrichment of mitochondrial pathways.

To further resolve the biological processes underlying these genotype-specific changes, DEPs were grouped into functional categories and compared across genotypes. While *Ctr1*^+/−^ and *Atp7a^T985I^* single mutants showed relatively modest and partially overlapping changes, the *Atp7a^T985I^*/*Ctr1*^+/−^ sciatic nerve exhibited a broader and higher-amplitude response across multiple functional categories, including oxygen/vascular processes, inflammation/glial activation, structural remodelling, proteostasis, and mitochondrial/neuronal pathways (Fig. 4E). This pattern supports a threshold model in which combined Atp7a dysfunction and reduced Cu availability drive coordinated proteomic remodelling in the PNS. To identify key drivers of this response, we next examined the most strongly altered proteins in *Atp7a^T985I^/Ctr1*^+/−^ sciatic nerve (Fig. 4F). This analysis revealed coordinated upregulation of proteins associated in oxygen handling and vascular responses (e.g. haemoglobin subunits), inflammatory and glial markers (e.g. Chil3, S100a9), and cytoskeletal organization (e.g. Krt24, Ctnna2), alongside changes in proteostasis (e.g. Ubqln1) In contrast, mitochondrial/neuronal proteins showed a mixed profile, including downregulation of cytochrome c oxidase subunit Cox7b and upregulation of the mitochondrial leucyl-tRNA synthetase Lars2.

## Discussion

In this study, we provide new insights into the molecular mechanisms driving axonal degeneration in X-linked dHMNX caused by *ATP7A* mutations, by establishing and characterizing a double mutant mouse model (*Atp7a^T985I^/Ctr1^+/−^*) with genetically restricted Cu access in the nervous system. The previous Atp7a dysfunction model (*Atp7a^T985I^)* reproduced selected molecular features of dHMNX, but did not develop robust motor or neuropathological phenotypes, limiting their value for interrogating disease pathogenesis [13]. By combining the *Atp7a^T985I^* mutation with heterozygous loss of the high-affinity copper transporter Ctr1, we generated a symptomatic model that recapitulates key aspects of the human disorder, including a modest motor phenotype and axonal loss.

*Ctr1^+/−^* and *Atp7a^T985I^/Ctr1^+/−^* animals both showed reduced Cu levels in the CNS and PNS (Fig. 1), however, the *Ctr1^+/−^* mice did not develop motor impairment, consistent with previous studies showing Cu reduction alone is insufficient to cause neuropathy [15]. Instead, the combined defects in Cu uptake (Ctr1 haploinsufficiency) and Cu distribution/export (*Atp7a^T985I^*) appear to push the system past a pathological threshold. In our study, reduced copper levels were measured in 45-day-old *Atp7a^T985I^/Ctr1^+/−^* mice. It remains unclear whether this early deficiency primes the system for later vulnerability, or if Cu levels remain persistently low throughout life. As Cu homeostasis may partially adapt or stabilize over time, it would be valuable to reassess Cu concentrations at later time points. Such data would help determine whether the observed neurodegeneration is driven by chronic defects in Cu uptake and distribution or reflects a delayed consequence of early-life copper-associated defects. In addition, as Cu levels were measured in whole sciatic nerve by ICP–MS, our data do not resolve cell type—specific or subcellular Cu distribution. Given the cellular complexity of peripheral nerve, it is therefore possible that total tissue Cu does not fully reflect Cu availability within motor axons or other vulnerable compartments. Altered intracellular Cu distribution or compartmentalization may thus also contribute to the observed phenotype.

Our behavioural tests for motor performance showed a modest motor phenotype in the *Atp7a^T985I^/Ctr1^+/−^* mice, with reduced endurance on the wire hanging test by six months of age (Fig. 2), an effect not observed in *Atp7a^T985I^* single mutants, indicating that the combination of reduced Cu uptake and impaired ATP7A function synergistically compromised neuronal health. While genotype-specific differences in grip strength were evident at 6 months, this was not the case at 12 months, where all genotypes showed similarly poor performance. This likely reflects a “floor effect” due to age- and weight-associated declines in hanging capacity across all groups, which can mask underlying functional differences. Such compression of performance metrics is a well-recognized limitation of strength and endurance tests in older or heavier mice [16], reinforcing the value of the 6-month timepoint as a more sensitive window for detecting genotype-dependent phenotypes.

Histopathological analysis is consistent with the length-dependent, distal axon degeneration in dHMNX [9]. The statistically significant reduction observed in the small-calibre myelinated axons in sciatic nerve from the Cu-restricted *Atp7a^T985I^* mice (Fig. 3), underscores the vulnerability of these fibres to Cu-dependent disruption and support the concept that adequate Cu availability is essential for maintaining peripheral axonal integrity. Interpretation of these findings should, however, be made with consideration of potential confounders in axon size distribution. While we report a reduction in the number of axons within the 2–3 µm diameter range, this could reflect either selective loss of small-calibre axons or a calibre shift due to axonal atrophy, whereby fibres fall into smaller bins rather than being lost entirely. Without complete axon diameter distribution plots or morphometric parameters such as g-ratio and myelin thickness, we cannot definitively distinguish between these possibilities. Future ultrastructural or high-resolution morphometric analyses would be valuable to resolve whether the observed phenotype primarily reflects axon loss, remodelling, or demyelination/remyelination processes.

Our proteomic analysis, strategically positioned at 12 months of age to capture late-stage pathway signatures, provides strong molecular support for a two-hit threshold model of disease. Given the genetically defined nature of the model and the aim to capture potentially subtle proteomic shifts underlying axonal vulnerability, we employed unadjusted *P*-values (*P* < .05) combined with a fold-change cutoff (±1.2) to maximize sensitivity. We acknowledge that this approach increases the potential for false positives in the context of large-scale proteomic datasets. Accordingly, our analysis should be interpreted as discovery-oriented, aimed at identifying coordinated biological patterns rather than individual protein-level changes in isolation. Importantly, the robustness of our conclusions is supported by the magnitude and consistency of the observed effects, including the marked increase in DEPs in the Cu-restricted *Atp7a^T985I^*/*Ctr1*^+/−^ mice (360 DEPs) compared to the relatively modest changes in *Ctr1^+/−^* and *Atp7a^T985I^* single mutants (19 and 47 DEPs, respectively), as well as the convergence of these changes across multiple biologically coherent functional categories. Together, these features argue against random signal and support a model in which copper restriction and ATP7A dysfunction synergistically drive widespread proteomic remodelling in the PNS.

The overwhelming upregulation of proteins in the double mutant further indicates a strong stress or adaptive response, consistent with the onset of axonal pathology. In depth analysis of the proteomic profile of *Atp7a^T985I^/Ctr1^+/−^* sciatic nerve supports a coordinated, multi-system response consistent with adaptive remodelling under conditions of copper restriction. The prominent upregulation of oxygen/vascular proteins (including multiple haemoglobin subunits and carbonic anhydrases) suggests altered oxygen handling and potential vascular or hypoxic stress. Concurrent increases in inflammatory and glial markers together with cytoskeletal and structural proteins point to active tissue remodelling and cellular stress responses. In parallel, changes in proteostasis-related proteins indicate engagement of stress adaptation pathways.

The heterogeneous response found of proteins engaged in copper-dependent and mitochondrial pathways (Fig. 4D-F) provide a mechanistic link to disease pathogenesis. In particular, reduced abundance of cytochrome c oxidase subunits (Cox7b, Cox6b1) suggests impaired mitochondrial respiratory chain function, consistent with the essential role of Cu in cytochrome c oxidase activity. This is of particular interest given that mutations in *COX6A1* cause a recessive form of Charcot-Marie-Tooth (CMT) disease [17], highlighting the sensitivity of peripheral nerves to disruption of mitochondrial respiratory chain components. These findings are suggestive of the contribution of mitochondrial dysfunction to the dHMNX phenotype and are consistent with our previous observations in iPSC-derived motor neurons from a patient harbouring the *ATP7A* p.T994I mutation [12]. In contrast, upregulation of Lars2 in the Cu-restricted *Atp7a^T985I^* mice may reflect a compensatory response aimed at maintaining mitochondrial protein synthesis under conditions of metabolic stress. Taken together, these findings support a model of bioenergetic stress rather than uniform mitochondrial activation. Disrupted Cu homeostasis may impair oxidative phosphorylation while simultaneously triggering compensatory responses in mitochondrial maintenance pathways. Given the high energetic demands of long peripheral axons, such imbalance between energy production and compensatory adaptation may contribute to axonal vulnerability in dHMNX.

Functional studies of the *ATP7A* p.T994I pathogenic variant have shown that this substitution disrupts interaction of the Cu transporter with p97/VCP [18]. Human p97, also known as valosin-containing protein (p97/VCP), is a central regulator of proteostasis, with key roles in protein quality control through the ubiquitin-proteasome system (UPS) and autophagy [19]. Consistent with this broad cellular importance, dominant missense variants in p97/VCP are implicated in multi-system proteinopathies including inclusion body myopathy in early-onset Paget disease and frontotemporal dementia (IBMPFD) [20], familial amyotrophic lateral sclerosis (fALS) 21] and have also been reported in an autosomal dominant form of CMT2 [22]. Although p97/VCP itself was not detected as a DEP in our TMT dataset, we observed selective dysregulation of multiple proteins linked to cellular proteostasis specifically in Cu-restricted *Atp7a^T985I^* mice. In particular, ubiquilin-1 (a protein in humans encoded by the *UBQLN1* gene) was significantly increased relative to wild-type controls (2.3-fold; log2FC = 1.2). UBQLN1, a protein that acts as a bridge between polyubiquinated proteins and the proteasome has been associated with Brown–Vialetto–Van Laere syndrome [23], and implicated in motor neuron biology, including recent reports identifying it as a p97/VCP interacting partner in SOD1–ALS patient iPSC-derived motor neurons. While these findings are exploratory and will require orthogonal validation, the increased abundance of ubiquilin-1 together with the proteasome/UPS associated changes, supports the idea of proteostasis disruption in peripheral nerve is a downstream consequence of impaired ATP7A-dependent copper handling, and may contribute to the emergence of the dHMNX phenotype in the *Atp7a^T985I^/Ctr1^±^* model.

Our Cu-restricted *Atp7a^T985I^*model provides a clinically relevant platform for dissecting Cu-dependent mechanisms that preserve axonal integrity and drive degeneration. Although the behavioural phenotype is modest, this likely reflects the early stage of disease captured in this model. In humans, ATP7A-associated distal motor neuropathy typically presents in adulthood and progresses slowly over decades [9, 10], whereas the shorter lifespan of mice may limit the emergence of more overt functional deficits. Species-specific differences in axonal resilience and compensatory capacity, may further explain why molecular and histopathological changes are more pronounced than motor impairment in the mouse model [24]. Our findings are consistent with a model in which peripheral nerve integrity is maintained within a relatively narrow window of copper availability, beyond which compensatory mechanisms fail and axonal pathology emerges. In this context, factors influencing systemic copper homeostasis, including dietary copper intake and metabolic state, may modulate disease penetrance or progression. The Cu-restricted *Atp7a^T985I^*model provides a tractable platform to test therapeutic hypotheses, including whether restoring copper availability, stabilizing ATP7A function with pharmacological chaperones, or enhancing axonal energy metabolism can ameliorate peripheral nerve pathology. Collectively, these findings deepen mechanistic insight into dHMNX and highlight the broader importance of Cu homeostasis as a determinant of neurodegenerative vulnerability and a potential therapeutic target.

## Material and methods

### Mice

All experiments conducted with mice were approved by The Sydney Local Health District Animal Welfare Committee (Protocol No 2018/017B—“The role of mutant ATP7A in distal hereditary motor neuropathy”) and were conducted in accord with the NSW Animal research Act Australian Code for the Care and Use of Animals for Scientific Purposes. The *Atp7a^T985I^* knock in mice were generated by Ozgene (Perth, Australia) as previously described [13]. Mice heterozygous for functional *Slc31a1* [14] were purchased from the Jackson Laboratory (strain #025 649) and a colony maintained by breeding with wild-type C57BL/6 J mice. Both colonies were maintained separately under standard housing conditions, which included *ad libitum* access to chow and water and a 12-h light/dark cycle as well as cardboard rolls for enrichment and tissue paper for nesting. To obtain experimental males for all genotypes used in this study, heterozygous *Slc31a1* males were crossed with heterozygous females for the *Atp7a^T985I^* mutation. The following genotyping primers were designed for genotyping the experimental animals for the *Atp7a^T985I^* mutation and to test the heterozygosity for *Slc31a1:*

*Atp7a* ^wt^_Fw: CCTACTTTCCCGTAAGTGACTCAT;
*Atp7a* ^wt^_Rev: AGTATGAAGGGAGAAACAGCTGAG;
*Atp7a^T985I^*_Rev: AGGATCTCCTGTCATCTCACCTT;
*Ctr1^wt^*_Fw*: TAATGTCCTGGTGCGTCTGA;*
*Ctr1^wt^*_Rev*: CAGCAGTAGATAAAAGCCAAGG;*
*Ctr1^mut^_Rev: TCACAGGTGACTTCCCAAGA*.
Two consecutives PCR reactions from digested toe clips were prepared for precise genotyping of the colony. Since dHMNX is X-linked, only male offspring were analysed in this study.

### Motor function assessment

*Rotarod test*. Mice were trained using a trial run during which the rotarod (LE8200 Accelerating Rota-Rod; SDR Scientific) was accelerated from 4 to 40 rpm over 300 s. After this acclimation, mice were timed for three runs on their ability to successfully continue running on the rod and the best performance was used to generate mean scores for each age group and genotype. A fall, a complete rotation, or completion of 300 s ended the run. Breaks (20 min) were given between each run. Data were collected using the Sedacom software provided with the instrument.

*Footprint analysis*. Mice were trained to walk along a narrow, straight corridor (80 cm in length) with a darkened goal box at the end. Forepaws and hindpaws were dipped in non-toxic red and blue paint, respectively, and the mice were allowed to walk across a sheet of white paper lining the floor of the corridor. From each mouse, at least three runs were recorded, and the most consistent footprint pattern was selected for analysis. Stride length (front and hind limbs) was measured as the distance between consecutive steps of the same paw. The hind paw angle was calculated by measuring the angle formed between the midline of the body and the line connecting the heel and third toe of each hind paw. Measurements were obtained from at least three consecutive steps per animal. Gait parameters were in a blinded fashion using digital images of the footprints.

*Hanging test*. Mice were placed on a 43-cm square wire mesh consisting of 12-mm squares of 1-mm-diameter wire and inverted. The latency to fall (i.e. the maximum hanging time) was measured. Each trial had a maximum time of 360 s.

### Tissue collection

Mice were killed for tissue collection as previously described. Briefly, the procedure involved deep anaesthetization using a cocktail of ketamine and xylazine, followed by transcardial perfusion using phosphate buffered saline supplemented with heparin, protease inhibitors, and phosphatase inhibitors. A small (∼5 mm) section of lumbar spinal cord was post-fixed in 4% (w/v) paraformaldehyde and then the remainder was snap frozen and stored at −80°C. Quadriceps, liver, kidney, heart, and brain were also excised from the animals, snap frozen, and stored at −80°C.

### Tissue copper quantification by ICP–MS

Total copper content was measured in tissues isolated from male mice at 45 days. Tissues were dissected, snap frozen, and stored at −80°C until analysis. Tissues were weighed and digested in 70% trace metal—grade nitric acid (Suprapur, Merck) at 95°C for 1–2 h until completely solubilized. Samples were then diluted to a final concentration of 2% HNO₃ using ultrapure Milli-Q water and analysed by inductively coupled plasma mass spectrometry (ICP–MS; Agilent 7700, Varian). Copper concentrations were normalized to wet tissue weight. All samples were run in technical triplicates, and data are presented as mean ± standard deviation (SD).

### Nerve histopathology

Sciatic nerves were cryoprotected in 30% sucrose and embedded in an optimal cutting temperature compound (Tissue-Tek), and 12-μm-thick transverse section were prepared for immunohistochemistry. Sciatic nerve tissue sections were permeabilized in 0.4% Triton X-100 and blocked in 4% normal donkey serum (NDS) with 1% bovine serum albumin for 1 h at room temperature followed by primary antibody for neurofilament (chicken, abcam, ab4680, 1:1000) at 4°C overnight. Alexa594 was used as secondary antibody from Jackson laboratory.

Myelinated axons were labelled by FluoroMyelin-green (1:500; Molecular Probe; a water-soluble fluorescent dye that selectively stains myelin’s lipid component) for 20 minutes at room temperature. Stained slides were then mounted using the antifade mounting medium with 4′,6-diamidino-2-phenylindole (DAPI) (H-1200).

Images were captured with a confocal microscope, Zeiss LSM700 or LSM710. For sciatic nerve analysis, 12–16 sections per animal were captured for nerve vesicles, nerve count, nerve diameter, myelinated axon numbers, and distribution of myelinated axon distributions using ImageJ (Fiji, Version 1.53c; National Institute of Health, USA). Statistical comparisons were performed with one or two-way analysis of variance (ANOVA) with Tukey’s multiple comparison test with post hoc test with GraphPad Prism (version 8.0) or Microsoft Excel. Probability values < .05, .01, or .001 were considered statistically significant.

### TMT-based quantitative proteomics

*Sample preparation and protein digestion:* Sciatic nerve tissues from 18 mice (across four experimental genotypes) were dissected, snap frozen, and submitted to the Australian Proteome Analysis Facility (APAF, Macquarie University, Sydney, Australia). Protein extraction was performed using S-Trap columns (Protifi, USA) following the manufacturer’s protocol. Tissues were lysed in 10% SDS, 100 mM triethylammonium bicarbonate (TEAB), pH 7.55, and homogenized by probe sonication. Disulfide bonds were reduced with 10 mM DTT at 56°C for 30 minutes and alkylated with 20 mM iodoacetamide in the dark for 30 minutes. Following acidification and binding buffer addition, samples were loaded onto S-Trap columns, washed, and digested with trypsin (1 µg/µL) for 3 hours at 47°C. Peptides were eluted sequentially using TEAB, 0.2% formic acid, and 50% acetonitrile with 0.2% formic acid, then vacuum dried and resuspended in 200 mM HEPES, pH 8.8.

*TMT labelling and fractionation:* Peptide concentrations were measured using the Pierce quantitative colorimetric peptide assay. Equal amounts of peptides were labelled using TMT 10-plex reagents (Thermo Fisher Scientific), with one pooled sample used as a reference in both runs. Labelling reactions were quenched with 5% hydroxylamine. Equalization of peptide loading across channels was confirmed by label check LC-MS/MS. Samples were pooled and cleaned using C18 solid-phase extraction (Sep-Pak, Waters), dried, and then subjected to high-pH reversed-phase fractionation using an Agilent 1260 HPLC system. A total of 96 fractions were collected and concatenated into 20 final fractions for LC-MS/MS analysis.

*LC-MS/MS data acquisition:* Peptides were separated on an EASY-nLC 1000 system (Thermo Fisher Scientific) using Halo-C18 trap and analytical columns (2.7 µm, 160 Å). Peptides were eluted over a 110-minute linear gradient and analysed on a Q-Exactive quadrupole-Orbitrap mass spectrometer. Full MS scans (350—1850 m/z) were acquired at 70 000 resolution, and the top 10 most intense ions were selected for HCD fragmentation (NCE 35), with MS/MS scans also acquired at 70 000 resolution. Only precursors with charge states + 2 to + 4 were selected. Dynamic exclusion was set to 90 s.

*Protein identification and quantification:* Raw data were processed using Proteome Discoverer (v2.1.0.81) with Mascot and SequestHT search engines against the UniProt mouse database (August 2018 release). Search parameters included: trypsin digestion with up to 2 missed cleavages, precursor mass tolerance of 20 ppm, fragment mass tolerance of 0.02 Da, static modification of carbamidomethyl (C), and dynamic modifications including oxidation (M), deamidation (N, Q), acetylation (N-term), and TMT6plex labels on K and N-termini. Protein, peptide, and PSM false discovery rates were all set to < 1%. A total of 4808 proteins were identified, and 3224 proteins were quantified across all biological replicates. Differential expression analysis was performed using pairwise t-tests with significance set at *P* < .05 and fold-change cut-off of ± 1.2

### Data analyses

All statistical analyses were performed using GraphPad Prism software. Significant differences between groups were determined using two-tailed *t* -tests or from two-way ANOVA with Tukey’s post hoc multiple comparisons test. The following statistical thresholds have been applied throughout the study: *P < .05; **P < .01; ***P < .001.

## Supplementary Material

mfag020_Supplemental_Files

## Data Availability

The data underlying this article are available within the article and its online Supplementary material. Additional data supporting the findings of this study will be made available by the corresponding author upon reasonable request.
