# Supplementary figures and images for: Copper restriction unmasks axonal degeneration in a mouse model of X-linked hereditary motor neuropathy
Source: Metallomics. 2026 Jun 5;18(1):mfag020. doi: 10.1093/mtomcs/mfag020 (PMC13309779; doi:10.1093/mtomcs/mfag020)

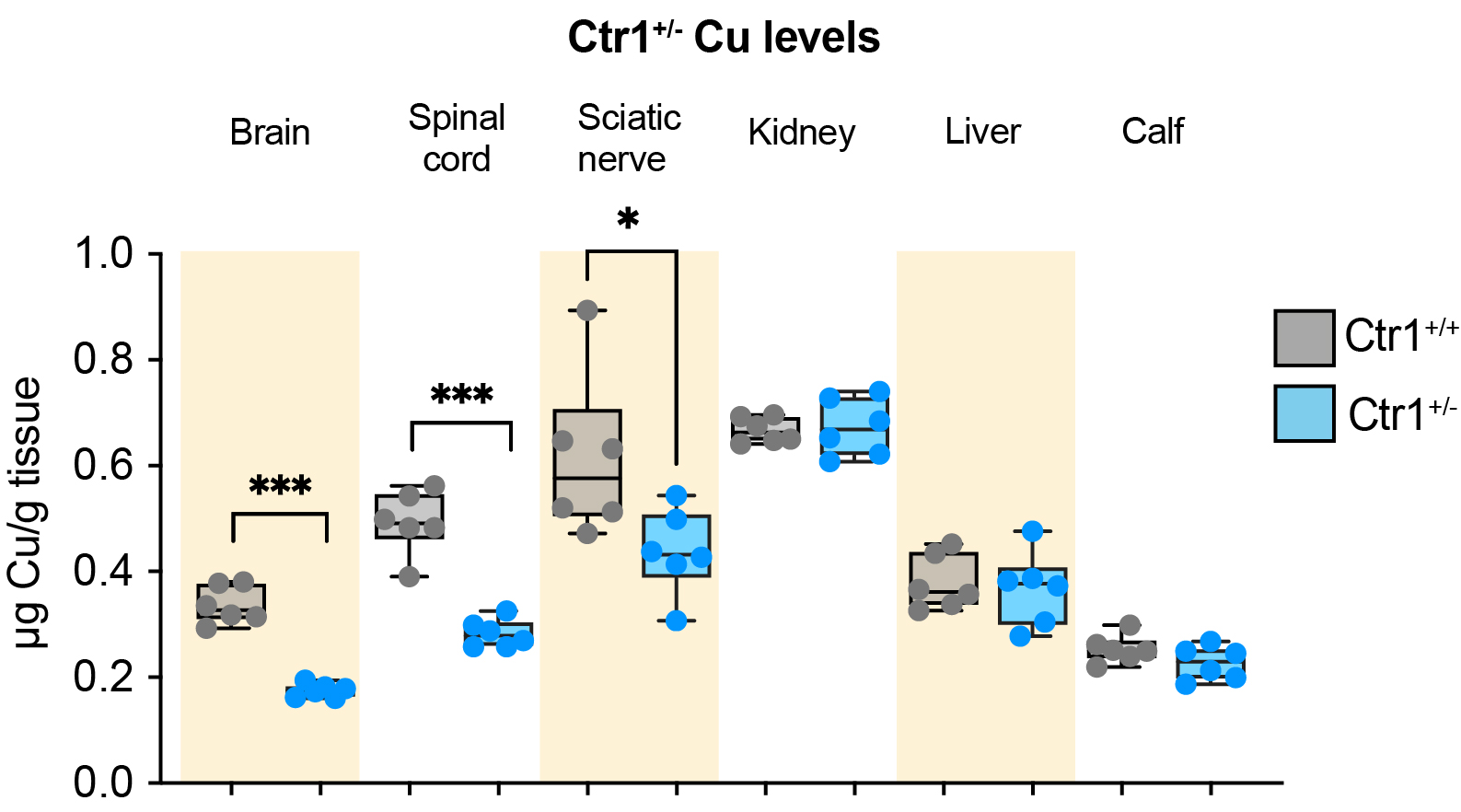

Supplement: mfag020_Supplemental_Files [file mfag020_supplemental_files.zip › SupplemFig1.jpg]

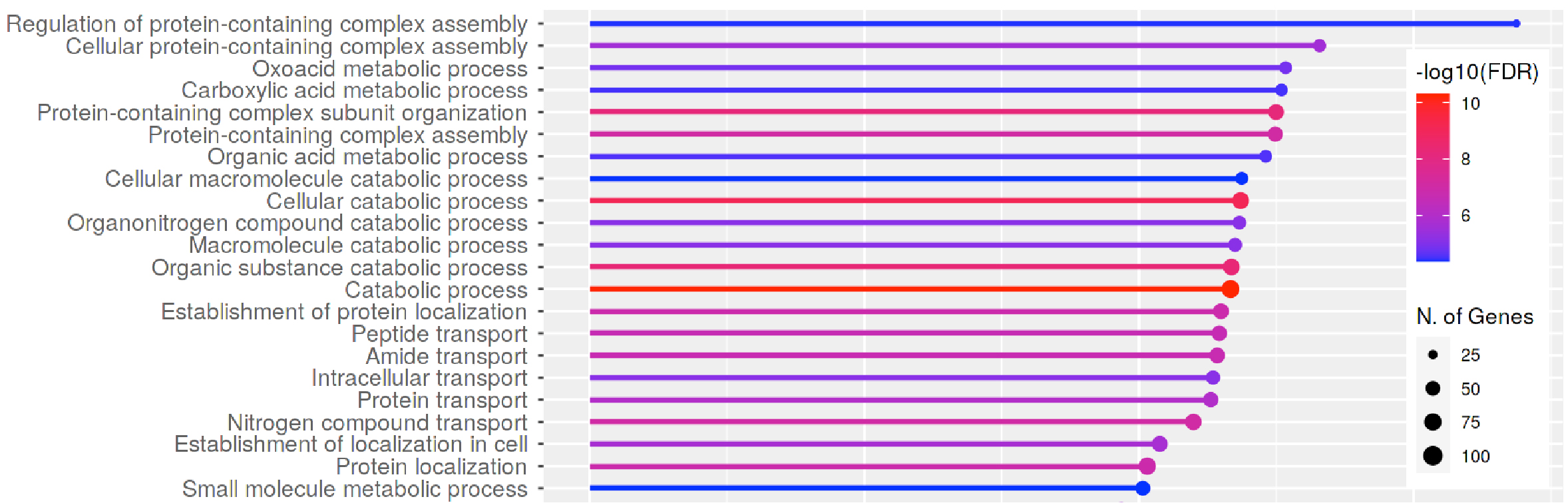

Supplement: mfag020_Supplemental_Files [file mfag020_supplemental_files.zip › SupplemFigure2_Revised.jpg]
